# Should I call psycho-oncology? Training nurses on psycho-oncological screening reduces uncertainties

**DOI:** 10.1007/s00432-023-04936-3

**Published:** 2023-06-08

**Authors:** Lara Dreismann, Karoline Schoknecht, Arndt Vogel, Tanja Zimmermann

**Affiliations:** 1 Department of Psychosomatic Medicine and Psychotherapy, Hannover Medical School, Hanover, Germany; 2 Department of Nursing Care Sector VI, Hannover Medical School, Hanover, Germany; 3 Department of Gastroenterology, Hepatology and Endocrinology, Hannover Medical School, Hanover, Germany

**Keywords:** Psycho-oncology, Screening, Interdisciplinary work, Cancer nursing, Training, Communication

## Abstract

**Purpose:**

Psycho-oncological screening is required to identify distressed patients and direct them to psycho-oncological care. In practice, screening procedure and related communication are still insufficient due to various barriers on the side of the medical team. The aim of this study is to evaluate the specifically developed training (OptiScreen training) on screening from nurses’ perspective.

**Methods:**

*N* = 72 nurses from visceral–oncological care at Hanover Medical School received the 6-h training, which consisted of three modules and targeted topics around screening, psycho-oncology and communication. The training was evaluated using a pre- and post-questionnaire assessing screening knowledge, uncertainties and further satisfaction outcomes.

**Results:**

Personal uncertainties were significantly reduced by the training (*t*(63) = − 13.32, *p* < .001, *d* = 1.67). General satisfaction with the training was achieved (62.0–98.6% satisfied with the training elements). Feasibility (69%) and general acceptance (94.3%) for the training were rated positively.

**Conclusion:**

The nurses rated the training as useful to reduce personal uncertainties regarding the screening process. Acceptability, feasibility and satisfaction with the training from the nursing perspective were achieved. The training contributes to minimizing barriers to inform about psycho-oncology and to recommend appropriate support services to patients.

**Supplementary Information:**

The online version contains supplementary material available at 10.1007/s00432-023-04936-3.

## Introduction

A cancer diagnosis is a high risk for experiencing a variety of psychosocial stressors. Approximately 50% of patients are significantly mentally distressed (Mehnert and Lehmann-Laue 2018), with 66% reporting clinically significant distress during acute hospitalization (Peters et al. 2020). The nursing team often provides comfort (Mick 2008) and plays a key role by providing continuous and close contact with patients (Dautel 2015; Musiello et al. 2017). However, it cannot provide comprehensive psychosocial support and often assumes a referral role to other treatment groups such as psycho-oncology (Morehouse 1997). This raises the question of what criteria are used to refer patients to psycho-oncology. Whilst nurses may tend to underestimate the psychological distress of their patients (Mitchell et al. 2008), there is also a risk of giving too much weight to certain signs of stress (e.g. patients crying). For this reason, screening instruments for the targeted identification of mental distress have been well established in practice for many years (Carlson and Bultz 2003). The National Institute for Health and Care Excellence recommends that nurses capture patients’ psychological distress (Gysels et al. 2004). The National Comprehensive Cancer Network (NCCN 2003), whilst not specifying a precise responsibility, also calls for routine screening. However, most nurses are not well informed about screening tools and their use. In addition, there is often a lack of information about psycho-oncological services as well as the effectiveness of psycho-oncological interventions (Mitchell et al. 2011; Neumann et al. 2010). Another typical hurdle in screening practice is the current workload of nurses. Often, screening is seen as an additional task and burden, especially when it is newly implemented. Yet, nurses are often responsible for distribution and collection of the screening questionnaire without being trained for it. Yet, they are the ones who get asked by patients about screening or psycho-oncological services. Without doubt, they also have a wide range of clinical impressions regarding the psychological distress of patients and are confidants, so that their recommendation for support services is particularly valued by patients. Since nursing and oncology nursing education curricula are up to the different regions/countries, psycho-oncological contents might vary. As pointed out in a previous study with nurses from different German regions and training levels, psycho-oncology and screening are not included in the education catalogue (Dreismann et al. 2021b). Experts and even patients might argue that a basic training on psycho-oncological topics should be mandatory for nurses that work in oncology. Training seems useful for nurses no matter what level of clinical experience or whether they already received oncology nursing training or not (Dreismann et al. 2021b). In order to integrate psychological distress more strongly into the daily treatment routine, there are calls to record psychological distress daily as a sixth vital sign (Holland & Bultz 2007). To achieve this, barriers need to be addressed (Dilworth et al. 2014) such as uncertainty amongst nurses to ask patients about psychological distress or to inform them about psycho-oncological support services (Dreismann et al. 2021a). A review found that training strategies are needed to resolve practitioner barriers, such as unclear referral to psycho-oncology, communication skills, acceptance and need for psycho-oncology (Dilworth et al. 2014).

For this reason, the OptiScreen-Study "Optimized psycho-oncological care through an interdisciplinary care algorithm-from screening to intervention" (Zimmermann et al. 2023) developed a training (OptiScreen-Training) on psycho-oncological screening for nurses, based on expert interviews with nurses (needs and hurdle analysis) (Dreismann et al. 2021a; Dreismann et al. 2021b), workshops with psycho-oncologists, current literature and research. Previous trainings, e.g. on recognition of psychological distress after initial diagnosis (Fukui et al. 2009) or increase of self-confidence regarding the communication of psycho-oncological services, show that nurses' detection of distress in patients was improved, also with regard to participant satisfaction (Kubota et al. 2015). There is also evidence that communication training can reduce feelings of insufficiency or emotional overwhelm in nurses (Bowles et al. 2001). In a review, improvements in self-efficacy, self-confidence, communication skills and greater awareness of psychological distress were achieved in 19 of 21 studies by communication training of oncology staff (Sheldon 2005). In addition, nurse training also has positive effects on patients. Nurses trained to address patient’s emotional problems were able to reduce emotional distress (Allard 2006) and strengthen coping strategies (Fukui et al. 2008). A Cochrane Review on communication training provides evidence of effectiveness in communication skills, information transfer and support skills (Moore et al. 2013). To date, there is no evaluated training that addresses both psycho-oncology screening and communication in the screening process, despite undoubted interest on the part of nurses to participate (Dreismann et al. 2021b). Ideally, a training evaluation according to Kirkpatrick and the hierarchical model pursues the goal of mapping four outcome levels: 1. Reaction, 2. Learning, 3. Behaviour, 4. Results (Kirkpatrick 1994). A systematic processing of descriptive and evaluative information of a training is necessary to classify further modifications and future implementations (Goldstein and Ford 2002). Therefore, the present study aims to evaluate satisfaction, usefulness, feasibility and acceptance of the OptiScreen training from the nurses' perspective.

## Methods

### Study design and procedure

**Fig. 1:**
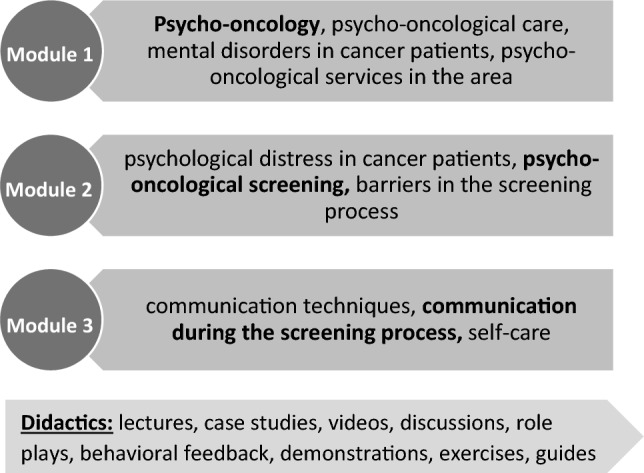
Contents of the optiscreen-training

The OptiScreen-Training for oncology nursing was developed as part of the OptiScreen-Study. The study aim was to design a pilot trial in order to examine feasibility and design of such a training (Zimmermann et al. 2023). The training consists of three topic modules, which can be delivered either on three single appointments of 1.5–2 h each or on a full training day of 4.5–6 h. In the present study, the training was conducted on three dates. The first module "Psycho-oncology and Psychological Disorders in Cancer Patients" targeted the three most common mental disorders amongst cancer patients: depression, anxiety and adjustment disorder (Mehnert et al. 2014; Mitchell et al. 2011) and psycho-oncological care structures (see Fig. 1). Module 2 "Psychological Distress and Screening Methods" introduced the screening tool that is used in this hospital, which is the distress thermometer (DT) including the problem list (Roth et al. 1998). Further examples of other screening tools were mentioned and listed in the training material as well. The third module "Communication in the Screening Process and Self-care" included videos and role-plays on different situations during the screening process. In addition, the module gave instructions for different self-care exercises, which fit the everyday life of nurses such as a ritual to end one’s work day and an exercise to distance and refocus after experiencing a burdening situation (5–4-3–2-1 coping technique). The topic modules include both theory-oriented parts for knowledge transfer and behavioural orientation with opportunities for practical exercise. The concept is based on theory- and literature-driven preliminary considerations, a qualitative needs and hurdle analysis through interviews with nurses (Dreismann et al. 2021a; Dreismann et al. 2021b) and a workshop with psycho-oncologists. Conceptualization is based on the "five first principles of instruction" and includes activation of prior knowledge, demonstration of desired behaviour, problem-solving orientation, applicability and transfer to everyday work (Merrill 2002). Participants consented by participating. Data were analyzed anonymously. Positive ethic vote has been received from Hannover Medical School (8478_BO_K_2019). A detailed training manual and all materials (in German) are available upon request and the detailed concept was published (Dreismann et al. 2023).

The OptiScreen-Training aims at a positive change in the handling of psycho-oncology screening and the transfer of information about psycho-oncological support from nurses to their patients. At the same time, personal barriers are to be reduced, and interdisciplinary exchange with the psycho-oncological team is to be promoted. The training alternates between lectures, case studies, discussions, video examples, role-plays in groups of two and practical exercises.

Both before and after the training, the nurses completed an evaluation form. An anonymous return of these forms was given since nurses used their own personalized code. The mean time difference between the pre- and the post-evaluation was *M* = 19.23 days (*SD* = 6.34).

### Participants

72 nurses participated in OptiScreen-Training from January to March 2021 at Hanover Medical School. Inclusion criteria involved working in the visceral oncology centre as nurses or case managers (with previous education in nursing or paediatric nursing, multiple professions/education could be stated). There were no exclusion criteria, as the entire team attended as mandatory training during working hours. Sixty-two were female, and most participants were between 20 and 64 years of age (see Table 1). Ninety-three per cent had no residency training in oncology and have worked in visceral oncology for about 10 years (*M* = 9.77, *SD* = 5.00; range: 0–38). Due to shift changes and sick leave, a total of 2 persons did not complete module 1, but only module 2 and 3.

**Table 1:** Demographic data of participants (*N* = 72)

Variables	*n*	%
Socio-demographics		
Female	62	86
Male	10	14
Mean age in years (standard deviation, range)	42.56 (11.66; 20–64)	
Profession		
Nurse	60	92
Paediatric nursing	3	4
Nursing assistant	1	2
Medical assistant	1	2
Case management	5	7
Oncology nursing	5	8
Previous training/continued education in psycho-oncology		
Yes	4	6
No	56	86
Unknowing	5	8
Work area		
Nursing	65	93
Case management	5	7
Work model		
Full-time	45	69
Part-time	20	31

*Note. N* partly reduced

### Measures

Sociodemographic data, general work conditions and stresses of the current work activity were self-reported by the participants.

#### The optiscreen-evaluation-questionnaire

The questionnaire was developed specifically for the evaluation of the OptiScreen-Training taking the hierarchical evaluation model into account (Kirkpatrick 1994). The pre-evaluation consists of five scales, the post-evaluation of eight, whilst some scores can be compared before and after the training (See Appendix 1). Questions can be answered on a five-point Likert scale, unless otherwise indicated. Higher scores represent higher satisfaction. The questionnaire still needs to be validated but Cronbach’s α in the current sample ranged between *α* = 0.75–0.89).

#### The training evaluation inventory (TEI)

(Ritzmann et al. 2014): The TEI is well validated and can be used to evaluate trainings, regardless of the content. After the OptiScreen training, it was used to complete the post-evaluation. The two dimensions “training outcomes” and “training design” each consist of five subscales and the 45 items can be answered on a five-point Likert scale (see Appendix 2). High sum values represent a higher rating of the respective underlying dimension; in the case of the "Perceived Difficulty" scale, a high rating indicates satisfaction with the level of difficulty of the training. The internal consistency in the present study is *α* = 0.96.

### Statistical analyses

Statistical analyses were performed using SPSS 21 for Windows (IBM 2020). Means, standard deviations and frequencies were calculated for descriptive evaluation. Pre–post differences were calculated using paired *t *tests. All tests were two-sided, and the significance level was set at 0.05.

## Results

### Pre-evaluation

**Fig. 2:**
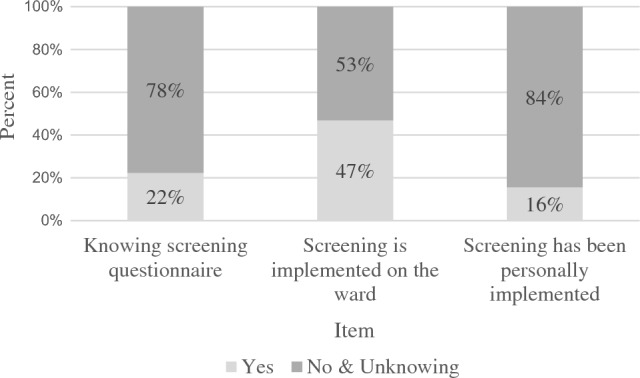
Screening experience in the pre-evaluation. *Note. N* = 64

The items of the scale screening knowledge of the pre-evaluation showed that the majority of nurses had no previous experience with psycho-oncological screening or its implementation in the daily routine of the ward (see Fig. 2). 53.3% had access to information material for patients e.g. flyer of psycho-oncological services.

The participants' agreement with various statements on psycho-oncology, its effectiveness and necessity, as well as the screening itself, are in the middle range rated by the participants in the pre-evaluation (see Table 2). The participants show the highest agreement values regarding the necessity of screening patients and the necessity of training on this topic.

**Table 2:** Attitude towards psycho-oncology in the pre-evaluation (before the training)

Item	*M*	SD	Mdn	Range	*N*
“Psycho-oncological support is effective and reduces psychological burden in patients.”	6.88	2.86	7.00	0–10	64
“The psychosocial screening should be implemented with all oncological patients.”	8.18	2.31	9.00	2–10	64
“Patients who are psychologically burdened currently receive a psycho-oncological council.”	5.70	3.14	6.00	0–10	64
“There is frequent discussion within the ward teams about the psychological burden of patients.”	5.86	3.33	7.00	0–10	63
„There is good contact and interdisciplinary conversation with the psycho-oncological team.”	4.51	2.92	5.00	0–10	63
“I am interested in speaking to patients about their psychological burden.”	7.20	2.90	8.00	0–10	64
“I believe trainings with a focus on psycho-oncological screenings are useful.”	8.09	2.85	9.00	0–10	64

*Note*. *Min* = 0 “completely disagree”; *Max* = 10 “completely agree”

A prior interest in the training topics seems to exist, as 85.9% consider psychological disorders/distress in cancer patients, 87.3% psycho-oncological care structures, 82.9% psycho-oncological screening, 89.1% communication with cancer patients and 82.8% self-care as topics within the training as "*quite useful*" or "*very useful*".

### Post-evaluation

#### Uncertainties

Table 3 shows that after training, nurses report significant improvement (*p* < 0.001) on the "uncertainties" scale items (criteria for psycho-oncology consultation, benefit of screening, explain screening questionnaire, inform about psycho-oncology, inform relatives about support offers, hand out flyers of psycho-oncology, know tasks of psycho-oncology and limit demanding relatives/patients). For the total scale, differences of the pre- (*M* = 2.69; *SD* = 0.93) and post-comparison (*M* = 4.11, *SD* = 0.67) were significant (*t*(63) = − 13.322, *p* < 0.001), with an effect size of Cohen's *d* = 1.67.

**Table 3:** Uncertainties rated by participants in the pre-post-evaluation

	Pre M (SD)	Post M (SD)	df	*t* test	Cohen ‘s d
Uncertainties/Insecurities					
Knowing about criteria for council	2.50 (1.14)	3.95 (0.90)	63	10.325**	1.29
Knowing about use of screening	2.81 (1.27)	4.23 (0.87)	63	8.817**	1.02
Explaining screening questionnaire to patients	2.40 (1.27)	4.29 (0.81)	61	10.680**	1.36
Informing patients about psycho-oncology	2.83 (1.22)	4.13 (0.86)	63	8.346**	1.04
Recommending support options to relatives	2.71 (1.26)	4.06 (0.93)	62	8.504**	1.07
Distributing flyers to patients	2.68 (1.28)	4.05 (1.02)	62	8.342**	1.05
Knowing about psycho-oncological tasks	2.81 (1.10)	4.39 (0.63)	63	11.524**	1.44
Handling/limiting demanding patients/relatives	2.66 (1.16)	3.81 (0.79)	61	7.199**	0.91

*Note. Min* = 1; *Max* = 5, **p < 0.001

### Evaluation of the training

#### Usefulness

As an indication of the functional benefit of the training, 87.5% of participants reported that after the training, they knew where to find the screening questionnaire to hand out to patients.

Regarding the usefulness of the respective training topics, none of the topics were rated as "*not at all useful*" or "*not very useful*". The topics psychological disorders/distress in cancer patients were rated as "*quite useful*" or "*very useful" by* 90.6%, psycho-oncological care structures by 82.1%, psycho-oncological screening by 93.7%, communication with cancer patients by 92.2% and self-care by 82.8%.

#### Satisfaction

In the post-evaluation, mean values were assessed for both the TEI and the specially created satisfaction scales. A rating of "*I agree*" and "*I strongly agree*" were scored as an indicator of satisfaction with each dimension (see Table 4). Participants showed satisfaction across all subscales ranging from 62.0 to 98.6%.

**Table 4:** Participant satisfaction with the training (post-evaluation)

Dimensions	Satisfied or very satisfied %	*M* (SD)
Presenters	95.8	4.73 (0.36)
Method of presentation	87.3	4.54 (0.50)
Usefulness of training topics	85.9	4.42 (0.53)
Atmosphere	80.3	4.40 (0.48)
Knowledge acquisition	70.4	4.20 (0.55)
TEI Training outcomes		
Perceived fun	98.6	4.72 (0.42)
Perceived difficulty	97.2	4.69 (0.43)
Attitude	93.0	4.56 (0.56)
Knowledge acquisition	90.1	4.46 (0.50)
Perceived usefulness	90.1	4.61 (0.46)
TEI training design		
Activation of prior knowledge	90.1	4.48 (0.51)
Demonstration	87.3	4.53 (0.48)
Integration	76.1	4.31 (0.56)
Problem-based learning	74.6	4.31 (0.55)
Application	62.0	4.07 (0.68)

*Note*. *N* = 71, *Min* = 1; *Max* = 5; The training evaluation inventory (TEI): 1 = “*I disagree*”, to 5 = “*I strongly agree*”

#### Feasibility and acceptance

Overall, 62 of 64 nurses (94.3%) would be likely (*“quite”/”very”/”somewhat”)* to recommend the training to colleagues, and 94.3% would like to receive regular training on psycho-oncology. 69.0% agreed or strongly agreed that the training was feasible. There is no difference between the fear of additional workload due to the training (*M* = 2.97; *SD* = 1.31) and the evaluation of the current additional workload after the training (*M* = 2.68; *SD* = 1.23; *t*(62) = 1.494, *p* = 0.140).

## Discussion

The aim of this study was to evaluate a training course for nurses on psycho-oncological screening with the aim of improving the identification of psychologically distressed patients and their referral to psycho-oncology. The OptiScreen-Training was well accepted and satisfactorily evaluated from the nursing perspective and closed existing knowledge and experience gaps on psycho-oncological screening. The usefulness of the content for nurses reached high agreement rates, which completed the reaction level of the evaluation model and is a predictor for the actual application in daily practice (Axtell et al. 1997; Kirkpatrick 1994). The training was rated as feasible and as integrable into everyday clinical routine. The rather lower feasibility rating compared to the satisfaction and acceptance levels of the training might be due to the timing of the training in this study. Each module took place after the early/before the later shift due to the requests in the expert interviews and the head nurses (Dreismann et al. 2021b). Participants rated this timeslot poorer due to their work load on the same day even though it was appreciated to combine it with going into work the same day. The training does not seem to have caused any additional effort in the daily clinical routine of the nursing staff. Possibly, the knowledge about the specific screening procedure as well as key phrases for practice led to a reduction of the previous barrier of (cognitive) effort. The lowest level of satisfaction was in the application and practical exercises. This could be due to the fact that the post-evaluation took place directly after the training and therefore an application was not yet possible. With regard to practical exercises during the training, inhibitions to perform role-plays were evident. Another reason might be the lack of integration in the daily nursing service catalogue which is still pending.

The results of the needs and hurdle analysis beforehand can be confirmed (Dreismann et al. 2021b). According to that analysis the evaluation shows that attitude towards psycho-oncology and interest in the training approved to be positive, confirming the motivation to participate as well as the interest of the nursing staff in patients’ mental health (Dautel 2015). Thus, the training is well adapted to the learning needs of nursing, thereby promoting transfer to work reality (Gaudine and Saks 2004). Analogous to other studies on communication trainings (Fukui et al. 2009; Sheldon 2005), the OptiScreen training led to a reduction of uncertainties regarding the screening or information process with large effect sizes and at the same time strengthen the nurses' confidence in communicating with cancer patients. On the learning level, according to the evaluation model, results showed that learning goals were achieved. The evaluation of the behavioural level also shows immediate improvement (Kirkpatrick 1994). Barriers on the part of nursing staff were reduced, e.g. in informing patients about psycho-oncological services or in recommending psycho-oncology support for patients (Dilworth et al. 2014; Mitchell et al. 2008; Neumann et al. 2010). Nurses were empowered in their key role and uncertainties were reduced. The existing evidence that nurses are able to perform the screening process after instruction is supported by the evaluation (Musiello et al. 2017). By training and engaging nurses, clinics can meet the quality requirements for guideline-appropriate psycho-oncological screening by integrating interdisciplinary work and recognizing the importance of needs assessment (Stengel et al. 2021). However, the involvement of other treatment groups, such as physicians, remains essential. Advanced training for the medical team or the establishment of the topic of "screening" in ward rounds and case discussions could promote interdisciplinary communication. The effects of training and the importance of psycho-oncological care should be shared by the entire treatment team so that they can be applied and regarded as standard in the long term.

### Perspectives for future research

Future research is needed to examine the long-term impact of continuing education on clinical practice. Continuing medical education, in particular, emphasizes the need for ongoing education to maintain competencies (Sullivan et al. 2019). Small reminders in clinical practice in the form of newsletters, notices, postcards, e.g. "*Already screened today?*" could be helpful for this purpose. Additionally, a one-hour booster session (Sullivan et al. 2019) is planned one year after training, including further assessment of the everyday use of the screening to complete the behaviour and result outcome levels of the evaluation (Kirkpatrick 1994). The booster session will reflect on the daily application and discuss hurdles and specific communication examples, as everyday transfer is consistently cited as the greatest source of training failure (Baldwin and Ford 1988). In the OptiScreen-Study, this is ensured by a study nurse who accompanies the project and visits participating wards weekly after training to remind them of the screening or to coach them on challenges in communicating with patients. The study nurse approach uses peer supervision methods that have a positive impact on transfer (Bachkirova and Jackson 2011).

Another important factor is the collection of outcome data at patient level (Barth and Lannen 2011; Razavi et al. 2003), which is planned as a baseline and intervention survey of patients in the OptiScreen-Study (Zimmermann et al. 2023). This will show the extent to which acquired nursing competencies are transferred to everyday life and whether patients have been both screened and informed regarding psycho-oncology. It is expected that this will increase the utilization of psycho-oncological support (Carlson et al. 2012). The survey also assesses patient satisfaction with nurses’ communication and recommendation of support services, which in turn may affect treatment satisfaction (Lehmann et al. 2009). In this study, only staff from visceral–oncological inpatient wards were trained, which is a relevant setting since it is known that patients show elevated anxiety and depression levels prior to surgery of gastrointestinal tumours (Harms et al. 2023). Nevertheless, further studies should also include a control group and examine implementation in other oncology settings or work environments, such as outpatient clinics.

### Limitations

Outcome evaluation on the patient side is still pending, so the study only represents evaluation from the nursing perspective. Also, the OptiScreen-Evaluation-Questionnaire developed specifically for the evaluation has not yet been sufficiently validated, but shows good reliability. Additionally, the literature often criticizes that satisfaction with training does not automatically lead to transfer of learning or behaviour (Cheng & Hampson 2008). Due to the time limitation, practice and application of the content in the training design (role-playing, feedback on behaviour) remain limited. Another factor to consider is that the training was mandatory and during duty hours, which might cause a bias but was demanded by the experts (Dreismann et al. 2021b). Attitudes towards psycho-oncology in our sample were already positive before the training and might be biased due to the upcoming training as well. The training was adapted to fit the German system of nursing education and screening procedures and may differ from practices in other countries.

## Conclusion

In sum, this is the first study for nurses that includes training on psycho-oncology and screening. Nurses play a key role in supporting cancer patients, so the OptiScreen-Training can encourage this role and professionalize it through appropriate knowledge and training in screening and communication. Widespread implementation can both strengthen the competence of nurses and increase the likelihood that distressed patients will receive the psycho-oncological care they need.

## Supplementary Information

Below is the link to the electronic supplementary material.

Supplementary file1 (DOCX 17 KB)

## Data Availability

The data that support the findings of this study are available on request from the corresponding author. The data are not publicly available due to privacy or ethical restrictions.
