# Supplementary material for: Should I call psycho-oncology? Training nurses on psycho-oncological screening reduces uncertainties
Source: J Cancer Res Clin Oncol. 2023 Jun 8;149(12):10585–92. doi: 10.1007/s00432-023-04936-3 (PMC10423155; doi:10.1007/s00432-023-04936-3)
Supplement: Supplementary file 1 — Supplementary file1 (DOCX 17 KB) [file 432_2023_4936_MOESM1_ESM.docx]

**Appendix 1** Scales of the OptiEva-Questionnaire

| **Scale** | **No. Items** | **Min** | **Max** | **M** | **SD** | **α** | **Rating mode** | **Item example** |
| --- | --- | --- | --- | --- | --- | --- | --- | --- |
| *Scales of the pre-evaluation* | | | | | | | | |
| Screening knowledge | 5 | - | - | - | - | - | Yes/no/I don’t know | “Have you ever personally implemented the screening-questionnaire?” |
| Uncertainties^a^ | 8 | 1 | 5 | 2.67 | 0.94 | .83 | 1 = “not at all” to 5 = “very much” | “How secure do you feel in explaining the screening questionnaire to patients?” |
| Usefulness of topics^a^ | 5 | 1 | 5 | 4.40 | 0.68 | .84 | 1 = “not at all” to 5 = “very much” | “How you useful do you think the topic psycho-oncological care structures is within the training?” |
| Interest in training | 10 | 1 | 5 | 3.94 | 0.65 | .84 | 1 = “not at all” to 5 = “very much” | “How important is it to you to broaden your knowledge within the training?” |
| Attitude towards psycho-oncology | 7 | 0 | 10 | 6.59 | 1.96 | .77 | 0 = ”I completely disagree” to 10 = ”I completely agree” | “Psycho-oncological interventions are effective and reduce distress in patients.” |
| *Scales of the post-evaluation* | | | | | | | | |
| Uncertainties^a^ | 10 | 1 | 5 | 4.04 | 0.61 | .89 | 1 = “not at all” to 5 = “very much” | “How secure do you feel in explaining the screening questionnaire to patients?” |
| Usefulness of topics^a^ | 5 | 1 | 5 | 4.43 | 0.53 | .87 | 1 = “not at all” to 5 = “very much” | “How you useful do you think the topic psycho-oncological care structures was within the training?” |
| Atmosphere | 5 | 1 | 5 | 4.41 | 0.48 | .75 | 1 = “not at all” to 5 = “very much” | ”How satisfied were you with the atmosphere within the training group?” |
| Learning gains | 9 | 1 | 5 | 4.21 | 0.55 | .89 | 1 = “not at all” to 5 = “very much” | “How satisfied were you with learning something new?” |
| Method of presentation | 7 | 1 | 5 | 4.54 | .50 | .91 | 1 = “not at all” to 5 = “very much” | “How satisfied were you with training materials provided?” |
| Presenters | 6 | 1 | 5 | 4.73 | 0.36 | .86 | 1 = “not at all” to 5 = “very much” | “How satisfied were you with the competence of the trainers/ speakers?” |
| Feasibility | 5 | 1 | 5 | 4.10 | 0.63 | .84 | 1 = “not at all” to 5 = “very much” | “How feasible do you rate the time frame of the training?” |
| Acceptability | 4 | 1 | 5 | 3.60 | 0.61 | .50 | 1 = “not at all” to 5 = “very much” | “How likely is it that you recommend the training to colleagues?” |

*Note.* No. = Number of; α = Cronbachs alpha, ^a^= Both used in pre- and post-questionnaire

**Appendix 2** Scales of the TEI

| **Scale** | **No. Items** | **Min** | **Max** | **M** | **SD** | **α** | **Rating mode** | **Item example** |
| --- | --- | --- | --- | --- | --- | --- | --- | --- |
| *TEI Training outcomes* | | | | | | | | |
| Perceived fun | 3 | 1 | 5 | 4.72 | 0.42 | .90 | 1 = “I disagree” to  5 = “I strongly agree” | “The Learning was fun.” |
| Perceived difficulty | 4 | 1 | 5 | 4.69 | 0.43 | .86 | 1 = “I disagree” to  5 = “I strongly agree” | “The contents were comprehensible.” |
| Perceived usefulness | 4 | 1 | 5 | 4.61 | 0.43 | .83 | 1 = “I disagree” to  5 = “I strongly agree” | “Investing time in this training was useful.” |
| Knowledge acquisition | 3 | 1 | 5 | 4.46 | 0.50 | .78 | 1 = “I disagree” to  5 = “I strongly agree” | “I will remember the new themes well.” |
| Attitude | 3 | 1 | 5 | 4.56 | 0.56 | .62 | 1 = “I disagree” to  5 = “I strongly agree” | “I will apply what I learned to my day-to-day work.” |

| *TEI Training design* | | | | | | | | |
| --- | --- | --- | --- | --- | --- | --- | --- | --- |
| Problem-based learning | 6 | 1 | 5 | 4.30 | 0.55 | .81 | 1 = “I disagree” to  5 = “I strongly agree” | “First of all, problems were addressed, and by working on them, I consequently learned the themes.” |
| Activation of prior knowledge | 5 | 1 | 5 | 4.48 | 0.51 | .85 | 1 = “I disagree” to  5 = “I strongly agree” | “My previous experiences regarding the themes covered were addressed.” |
| Demonstration | 7 | 1 | 5 | 4.53 | 0.48 | .83 | 1 = “I disagree” to  5 = “I strongly agree” | “The learning objectives were achieved.” |
| Application | 5 | 1 | 5 | 4.07 | 0.68 | .87 | 1 = “I disagree” to  5 = “I strongly agree” | “In the training I had the opportunity to try out things which I should later implement in my work.” |
| Integration | 5 | 1 | 5 | 4.32 | 0.56 | .78 | 1 = “I disagree” to  5 = “I strongly agree” | “I know the importance of the individual themes for different situations.” |

*Note.* No. = Number of; α = Cronbachs alpha, TEI = The Training Evaluation Inventory, only used in the post-questionnaire
